# Metabolic syndrome is independently associated with improved overall survival to first-line therapy with immune checkpoint inhibitors in non-small cell lung cancer

**DOI:** 10.3389/fonc.2023.1134824

**Published:** 2023-05-12

**Authors:** Maroun Bou Zerdan, Prashanth Ashok Kumar, Dulce M. Barrios, Alanna Glidden, Dayana Nasr, Stephanie Niforatos, Ghanshyam Ghelani, Jennifer Leibovitch, Sandy Nasr, Binod KC, Mulham Ombada, Farzam Khokhar, Bhavya Poudyal, Jenish Bhandari, Myera Shahnawaz, Stephen Graziano, Seah H. Lim

**Affiliations:** ^1^ Department of Medicine, State University of New York Upstate Medical University, Syracuse, New York, NY, United States; ^2^ Division of Hematology and Oncology, State University of New York Upstate Medical University, Syracuse, New York, NY, United States

**Keywords:** metabolic syndrome, non-small cell lung cancer, immune checkpoint inhibitors, treatment outcome, chemoimmunotherapy

## Abstract

**Background:**

Many co-existing medical conditions may affect the outcome in patients treated with immune checkpoint inhibitors for advanced cancer. There is currently not any information on whether metabolic syndrome (MetS) impacts the clinical outcome in patients treated with immune checkpoint inhibitors (ICIs) for advanced non-small cell line cancer (NSCLC).

**Methods:**

We carried out a single-center retrospective cohort study to determine the effects of MetS on first-line ICI therapy in patients with NSCLC.

**Results:**

One hundred and eighteen consecutive adult patients who received first-line therapy with ICIs and had adequate medical record information for the determination of MetS status and clinical outcomes were included in the study. Twenty-one patients had MetS and 97 did not. There was no significant difference between the two groups in age, gender, smoking history, ECOG performance status, tumor histologic types, pre-therapy use of broad-spectrum antimicrobials, PD-L1 expression, pre-treatment neutrophil:lymphocyte ratio, or proportions of patients who received ICI monotherapy or chemoimmunotherapy. With a median follow-up of 9 months (range 0.5-67), MetS patients enjoyed significantly longer overall survival (HR 0.54, 95% CI: 0.31-0.92) (*p* = 0.02) but not progression-free survival. The improved outcome was only observed in patients who received ICI monotherapy and not chemoimmunotherapy. MetS predicted for higher probability of survival at 6 months (*p* = 0.043) and 12 months (*p* = 0.008). Multivariate analysis indicated that, in addition to the known adverse effects of use of broad-spectrum antimicrobials and the beneficial effects of PD-L1 (Programmed cell death-ligand 1) expression, MetS was independently associated with improved overall survival but not progression-free survival.

**Conclusions:**

Our results suggest that MetS is an independent predictor of treatment outcome in patients who received first-line ICI monotherapy for NSCLC.

## Introduction

Obesity is a major public health problem affecting many countries. In the mouse models, obesity is associated with lymph node atrophy (1) and reduced T-cell receptor (TCR) diversity (2). Dendritic cells in obese mice exhibit reduced T-cell stimulatory capacity (3). Obesity is also associated with reduced abundance of intestinal *Akkermansia muciniphila* (4, 5), that was previously found to be associated with inferior treatment outcome to immune checkpoint inhibitors (ICIs) in patients with advanced cancer (6, 7). Chronic inflammatory processes occur in obesity (8, 9). Although obese individuals are more susceptible to tumor development and poorer treatment outcome in certain cancer types, obesity has conferred protective effects in other cancers (10, 11). This “obesity paradox” (12) has been seen in patients with non-small cell lung cancer (NSCLC) treated with ICIs. However, while many studies showed improved tumor outcome in obese patients (13, 14), several other studies found either the lack of benefit or negative effects of obesity in these patients (15, 16). Recent studies identified a group termed “metabolically healthy obesity” (17, 18) and highlighted the heterogeneity of obesity. The conflicting data on the effects of obesity in patients treated with ICIs for advanced cancer may, therefore, be related to the heterogeneity associated with obesity.

Alongside obesity, the incidence of metabolic syndrome (MetS) is also increasing. Patients with MetS experience glucose intolerance, central obesity, hypertriglyceridemia, reduced levels of high-density lipoprotein (HDL) cholesterol, and hypertension (19). To make a diagnosis of MetS, at least three of these five criteria have to be met. Not unlike obesity, MetS is also associated with ongoing inflammatory processes (20). Patients with MetS are at increased risks for the development of Type 2 diabetes mellitus, cancer, and cardiovascular diseases (21).

Although there is an overlap between obesity and MetS, not all obese patients have MetS. In the present study, we explored the association between obesity, MetS, and survival in patients with advanced NSCLC treated first-line with ICIs. Our main objective was to investigate the association between obesity, MetS, and treatment outcomes from ICI monotherapy or chemoimmunotherapy within the same cohort of patients treated by the same group of medical oncologists in a single institution.

## Patients and methods

### Data collection

A retrospective cohort study was performed on all patients ≥ 18 years of age who had received either PD-1 inhibitors (nivolumab or pembrolizumab) or CTLA-4 inhibitor (ipilimumab) with or without combination chemotherapy for advanced NSCLC at Upstate University Hospital, Syracuse, New York during the period from January 1, 2016 to December 31, 2020. Patient demographics, smoking history, clinical data, cancer diagnosis, and treatment history were collected. Data on antimicrobial use within four weeks prior to the initiation of anticancer therapy and the names of the antimicrobial used were also collected. Broad-spectrum antimicrobials were defined as antibiotics that are effective against both Gram-negative and Gram-positive organisms. We chose the 4-week time point to evaluate the effects of antibiotics on clinical benefit and survival, because any intestinal microbiome changes due to antibiotics would last longer than 4 weeks. The study was conducted with Institutional Review Board exemption from State University Upstate Medical University Institutional Review Board.

### Definitions

Our primary endpoint was clinical benefits and overall survival (OS). Clinical Benefit to therapy were determined by reviewing and comparing the imaging modality (CT or PET) at baseline and after starting therapy. Tumor responses were classified as Complete Response (CR) if there was a total resolution, Partial Response (PR) if at least 50% reduction, and Stable Disease (SD) if there was no significant change (<20% enlargement) or reduction of <50% of the tumor mass. No response to treatment was given if there was progression on imaging. Clinical Benefit was defined as CR + PR + SD. RECIST criteria were not used for response evaluation in our institution outside the context of clinical trials.

Overall survival (OS) measured the time from start of therapy to death due to any cause and progression-free survival (PFS) the time from start of therapy to radiologic and/or clinical disease progression necessitating change of therapy. Clinical Benefit rate was defined at the proportion of treated patients based on their best response during therapy.

### Diagnosis of metabolic syndrome

The National Institute of Health (NIH) guidelines define MetS as having three or more of the following characteristics (19): 1. Large waist, at least 35 inches for women and 40 inches for men; 2. High triglyceride level of 150 mg/dL or 1.7 mmol/L, or higher; 3. Reduced level of high-density lipoprotein (HDL) cholesterol of less than 40 mg/dL or 1.04 mmol/L in men or less than 50 mg/dL or 1.3 mmol/L in women; 4. Hypertension, with blood pressure of 130/85 mm Hg or higher; and 5. Elevated fasting blood glucose of 100 mg/dL or 5.6 mmol/L or higher

Since waist circumference was not a clinical parameter readily available in the patient’s medical records, the following modified criteria was used for the diagnosis of MetS in this study: 1. Subjects with a diagnosis of MetS documented in the medical record was considered to have the disorder; and 2. In the absence of measurements for the waist circumference, it was assumed that patients with a body mass index (BMI) of 30 or higher would satisfy the NIH criteria for large waist given the high correlation between BMI and waist circumferences for either gender (22). BMIs in this study were calculated based on the weight and height of the patients just prior to starting lung cancer therapy.

### Statistical analyses

Cox proportional hazards regression analysis were carried out to determine the hazard ratios (HRs) for the following covariates in PFS and OS: Prior use of broad-spectrum antimicrobials, age, tumor histology, metabolic syndrome, neutrophil:lymphocyte (N:L) ratio, obesity, PD-L1 expression, and smoking history. Results were presented individually in forest plots and as survival at mean of co-variates.

We next divided the 118 patients into two groups, based on whether they fulfilled the criteria for a diagnosis of MetS or not. We next determined whether the survival advantage associated with MetS occurred in both patients treated with ICI monotherapy and with chemoimmunotherapy. To do so, we stratified the patients according to whether they received first-line ICI monotherapy or chemoimmunotherapy for NSCLC. Finally, we determined if obesity was associated with improved outcome to ICI therapy in our cohort of patients.

The Clinical Benefit rate, progression-free survival (PFS) and OS were calculated. Survival was plotted as a time-dependent covariate using the Kaplan-Meier method. Association of factors potentially predictive of Clinical Benefit was evaluated using the Chi-square tests. Differences in the various clinical and laboratory parameters were calculated as mean and compared using the Student’s t tests. A two-sided p value of < 0.05 was considered statistically significant.

## Results

### Patients

Between January 1, 2016 to December 31, 2020, a total of 712 patients were diagnosed with NSCLC at our institution. One hundred and eighty-four patients received first-line therapy with ICIs. However, only 118 patients were included for analysis because the other 66 patients did not have sufficient data in their medical record for the determination of their MetS status, treatment response or other clinical outcomes. To ensure that we did not select for those with different treatment outcomes, we compared the characteristics of the patients in these two groups. We did not find any significant difference in age, gender, ECOG score, tumor histologic type, therapeutic modality, PD-L1 expression, use of broad-spectrum antimicrobials, smoking history, and N:L ratio between the two groups of patients (**Table 1**), supporting the notion that the cohort of patients included in the analysis was representative of the entire population of patients.

**Table 1:** Comparison of the characteristics between the patients who were included in and excluded from the analysis due to availability of data for diagnosing metabolic syndrome.

Parameter	Complete dataset (n = 118)	Incomplete dataset (n = 66)	*p* value
Gender (F:M)	61:57	37:29	*n.s.*
Age (year) Median Range	66.5 43-86	66.5 23-88	*n.s.*
ECOG performance status 0 1 2 3	41 46 26 5	29 24 11 2	*n.s.*
Histologic type AdenoCa Squamous Ca Adenosquamous	90 21 7	51 14 1	*n.s.*
PD-L1 (%) Median Range	50 0-90	50 0-90	*n.s.*
Broad-spectrum antimicrobial use	32	11	*n.s*.
Therapeutic modality ICI only CIT	57 61	33 33	*n.s.*
Smoking (Y/N)	57/61	30/36	*n.s.*
N:L ratio Median Range	5.16 0.55-31.45	4.52 0.82-21.66	*n.s.*

(F:M, Female : Male; Ca, Carcinoma; PD-L1, Programmed cell death-ligand 1; ICI, Immune checkpoint inhibitor; CIT, Chemoimmunotherapy; N:L, Neutrophil : Lymphocyte). NS, not significant.

Ninety-one patients (77%) had adenocarcinoma, 20 (17%) squamous cell carcinoma, and 7 (6%) mixed adenosquamous carcinoma. There were 61 females and 57 males. Median age was 66 years (range 43-86), ECOG performance status 1 (range 0-3), and PD-L1 expression 50% (range 0-90%). Thirty-two (27%) patients received broad-spectrum antibiotics within four weeks of starting their first-line NSCLC therapy. Fifty-seven (48%) patients received ICI monotherapy and 61 (52%) chemoimmunotherapy. The therapy regimens used are shown in **Table 2**. Despite being initiated on therapy, 50 patients (42%) did not respond to the treatment and they experienced progression of disease (PD).

**Table 2:** Distribution of the various treatment regimens used in our cohort.

First-line treatment regimens administered to patients	
Regimen	Number (n)
Pembrolizumab only	55
Nivolumab only	1
Combination of nivolumab and ipilimumab	1
Pemetrexed/carboplatin/pembrolizumab	51
Paclitaxel/carboplatin/pembrolizumab	8
Pemetrexed/pembrolizumab	1
Carboplatin/gemcitabine/nivolumab	1

### Factors influencing treatment outcomes to immune checkpoint inhibitors

**Figure 1:**
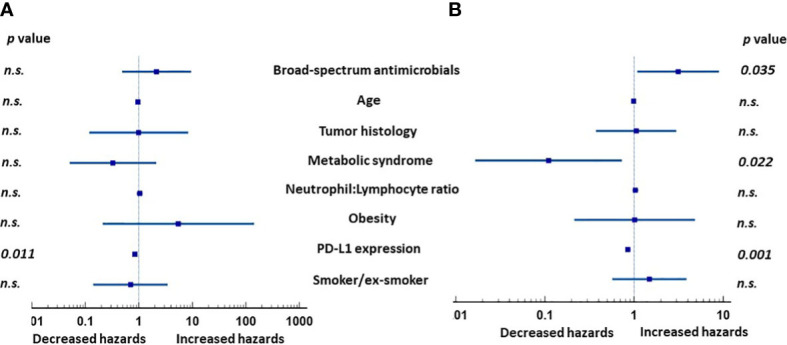
Hazard ratios (HRs) of clinical factors in multivariate analysis in patients with advanced lung cancer treated with either immunotherapy or immunochemotherapy. **(A)** Expression of PD-L1 was the only significant factor affecting progression-free survival. **(B)** In contrast, prior use of broad-spectrum antimicrobials, MetS, and PD-L1 expression were all independently associated with overall survival. NS, not significant.

We next determined the effects of the following a set of clinical parameters on the outcome of the entire group of analyzed patients: Prior broad-spectrum antimicrobial use, age, tumor histology, MetS, N:L ratio, obesity, PD-L1 expression, and smoking. None of the covariates, except PD-L1 expression, affected PFS of the patients in univariate analysis (**Table 3**). However, MetS (*p* = 0.02), lower N:L ratio (*p* = 0.02), and higher PD-L1 expression (*p* = 0.05) were associated with lower HRs. Multivariate analysis showed PD-L1 expression (*p* = 0.011) to be the only factor (**Figure 1A**) that affected PFS favorably. In contrast, MetS (*p* = 0.022) and PD-L1 (*p* = 0.001) expression were associated with lower risks and prior use of broad-spectrum antimicrobials (*p* = 0.035) a higher risk for overall survival (**Figure 1B**). **Table 4** shows the HRs for each of the covariates. These results suggest that, in addition to PD-L1 and prior use of broad-spectrum antimicrobials, MetS is an independent prognostic factor in patients with advanced lung cancer treated with immune checkpoint inhibitors +/- chemotherapy.

**Table 3:** Univariate analysis to determine hazard ratios associated with clinical characteristics on progression-free survival and overall survival.

Parameter	Progression-free survival		Overall survival	
	HR (95% CI)	*p* value	HR (95% CI)	*p* value
Prior antimicrobial use (Yes)	0.78 (0.35-1.75)	*n.s.*	0.93 (0.56-1.55)	*n.s.*
Increasing age	1.02 (0.99-1.06)	*n.s.*	1.01 (0.99-1.03)	*n.s.*
Histology (Adenocarcinoma or others)	1.22 (0.71-2.09)	*n.s.*	1.29 (0.91-1.82)	*n.s.*
Presence of MetS	0.48 (0.18-1.24)	*n.s.*	0.49 (0.25-0.94)	0.02
Increasing N:L ratio	1.23 (0.98-1.08)	*n.s.*	1.03 (1.01-1.06)	0.02
Presence of obesity	1.49 (0.64-3.44)	*n.s.*	1.47 (0.85-2.55)	*n.s.*
Higher PD-L1	0.99 (0.97-1.00)	*n.s.*	0.99 (0.98-1.1.00)	0.05
Smoking	1.56 (0.58-2.32)	*n.s.*	1.23 (0.79-1.91)	*n.s.*

(HR, hazard ratio; CI, Confidence interval; MetS, Metabolic syndrome; n.s., Not significant; N:L ratio, Neutrophil : Lymphocyte ratio; PD-L1, Programmed cell death-ligand 1). NS, not significant.

**Table 4:** Multivariate analysis of covariates.

	Progression-free survival			Overall survival		
	HR	95% CI	*p* value	HR	95% CI	*p* value
Broad-spectrum antimicrobial use (Yes)	2.14	0.49 to 9.45	0.314	3.12	1.09 to 8.97	0.035
Age (increasing age)	0.95	0.87 to 1.04	0.289	0.99	0.94 to 1.04	0.691
Histology (Adenocarcinoma or others)	0.99	0.12 to 8.29	0.992	1.05	0.37 to 2.97	0.921
Diagnosis of MetS	0.33	0.05 to 2.12	0.242	0.11	0.02 to 0.73	0.022
Neutrophil : Lymphocyte ratio (increasing ratio)	1.04	0.92 to 1.17	0.523	1.03	0.96 to 1.11	0.373
Presence of obesity	5.47	0.21 to 141	0.306	1.02	0.21 to 4.82	0.985
PD-L1 (higher %)	0.96	0.93 to 0.99	0.011	0.98	0.96 to 0.98	0.001
Smoking	0.70	0.14 to 3.46	0.660	1.48	0.57 to 3.86	0.422

### Effects of MetS on treatment outcomes

**Figure 2:**
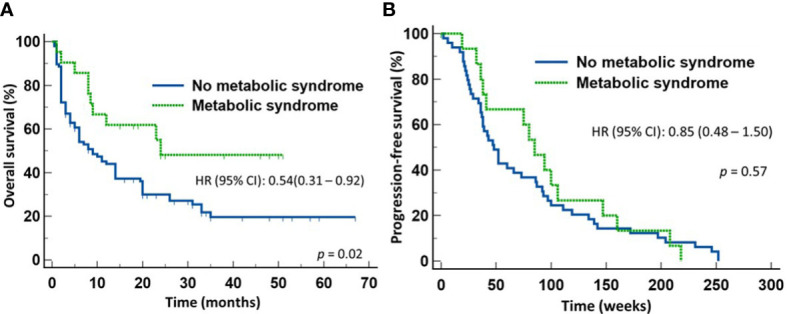
Outcome of patients with advanced non-small cell lung cancer treated first-line with either immune checkpoint inhibitors or chemoimmunotherapy. Patients with metabolic syndrome enjoyed improved overall survival **(A)** but not progression-free survival **(B)** compared to those without metabolic syndrome.

The results of the multivariate analysis led us to divide the 118 evaluable patients into two groups according to whether they had MetS or not to further evaluate the effects MetS had on treatment outcomes. Since the diagnosis of MetS requires at least three of the five criteria modified by us based on the NIH guidelines, only patients with a BMI > 30 and fulfilled at least two of the other four criteria, or those with BMI <30 but fulfilled at least three of the other four criteria were assigned the diagnosis of MetS. Based on these criteria, twenty-one (17.8%) patients were classified as having MetS and 97 (82.2%) non-MetS based on the available clinical characteristics. **Table 5** shows the clinical characteristics of the two groups. They were comparable in gender distribution, age, ECOG performance status, histologic types of the tumor, therapeutic modality, prior use of broad-spectrum antimicrobials, or PD-L1 expression. With a median follow-up of 9 months (range 0.5-67), the group of patients with MetS enjoyed significantly longer OS (median = 22 months) compared to those who did not have MetS (median = 9 months) (HR 0.54, 95% CI: 0.31-0.92) (*p* = 0.02) (**Figure 2A**). As expected from the results of the multivariate analysis, there was no significant difference in the PFS between the two groups (**Figure 2B**), suggesting that patients with MetS who were treated with first-line ICIs might have responded or tolerated better to second-line therapy when their disease progressed than those who did not have MetS. The probabilities of clinical benefits/disease-control (Complete Response + Partial Response + Stable Disease) were comparable between the two groups of patients (71.4% *vs* 54.6%; *p* = 0.22).

**Table 5:** Comparison of the characteristics between the patients with metabolic syndrome and patients without metabolic syndrome.

Parameter	Metabolic syndrome (n = 21)	No metabolic syndrome (n = 97)	*p* value
Gender (F:M)	10:11	51/46	*n.s.*
Age (year) Median Range	61 44-84	66 43-86	*n.s.*
ECOG performance status 0 1 2 3	8 6 5 2	33 40 21 3	*n.s.*
Histologic type AdenoCa Squamous Ca Adenosquamous	15 3 3	75 18 4	*n.s.*
PD-L1 (%) 0-50 51-100	62.5 37.5	54.5 45.5	*n.s.*
Broad-spectrum antimicrobial use	9	23	*n.s.*
Therapeutic modality ICI only CIT	10 11	47 50	*n.s.*
Smoking (Y/N)	7/14	50/47	*n.s.*
N:L ratio Median Range	7.2 2.03-20.82	5.3 0.55-46.79	*n.s.*

(F:M, Female : Male; Ca, Carcinoma; PD-L1, Programmed cell death-ligand 1; ICI, Immune checkpoint inhibitor; CIT, Chemoimmunotherapy; N:L, Neutrophil : Lymphocyte). NS, not significant.

### Metabolic syndrome affects treatment outcome only with immune checkpoint inhibitor monotherapy and not chemoimmunotherapy

**Figure 3:**
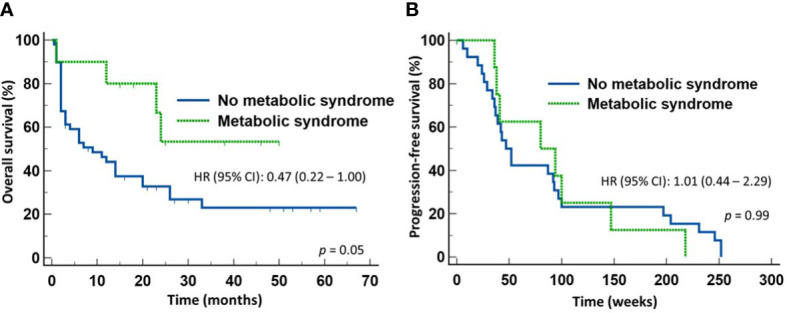
Outcome of patients with advanced non-small cell lung cancer treated first-line with only immune checkpoint inhibitors. Patients with metabolic syndrome enjoyed improved overall survival **(A)** but not progression-free survival **(B)** compared to those without metabolic syndrome.

**Figure 4:**
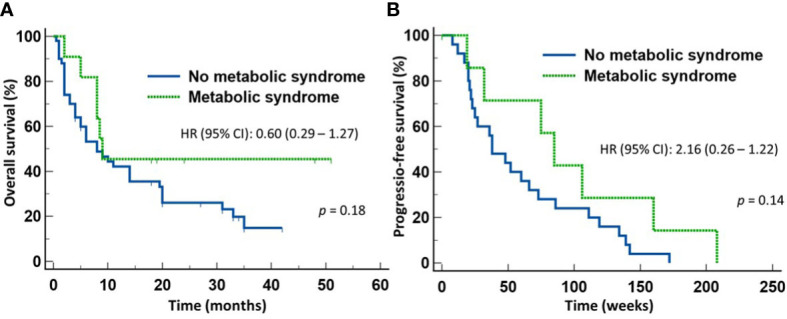
Outcome of patients with advanced non-small cell lung cancer treated first-line with chemoimmunotherapy. There was no statistical difference in either the overall survival **(A)** or progression-free survival **(B)** between those with metabolic syndrome or without metabolic syndrome.

We next determined whether the survival advantage associated with MetS occurred in both patients treated with ICI monotherapy and with chemoimmunotherapy. To do so, we stratified the patients according to whether they received first line ICI monotherapy or chemoimmunotherapy for NSCLC. Of the 57 patients who received only ICI monotherapy, 10 patients had MetS and 47 did not. There was no significant difference between the two groups in terms of gender distribution, age, ECOG performance status, histologic types of the tumor, or PD-L1 expression. However, 7/10 (70%) patients with MetS and10/47 (22.7%) of patients without MetS received broad-spectrum antimicrobials within four weeks of starting anti-NSCLC therapy (*p* = 0.005). Despite the high proportion of patients with MetS received broad-spectrum antimicrobials, patients with MetS still enjoyed significantly longer OS (median not reached) (HR 0.47, 95% CI: 0.22-1.00) (*p* = 0.05) (**Figure 3A**) but not PFS (**Figure 3B**) when compared to those without MetS (median = 8 months). In contrast, there was no significant difference in the OS (**Figure 4A**) or PFS (**Figure 4B**) in patients who were treated with first-line chemoimmunotherapy, whether the patients had MetS or not. The disease-control rates were also comparable between the two groups of patients irrespective of the treatment modality.

To dissect the effects of the prior use of broad-spectrum antimicrobials from those due to MetS in patients treated with ICI monotherapy, we performed a multivariate analysis of the two covariates for OS at two time points. MetS and not prior use of broad-spectrum antimicrobials predicted OS at six months (*p* = 0.043) and at twelve months (*p* = 0.008).

### Obesity and treatment outcome

Twenty-nine (24.5%) patients had BMI ≥30 and fulfilled the criteria for obesity. Four patients who were classified as having MetS had BMIs <30, and 12 patients with a BMI >30 did not have MetS. There was no significant difference between the two groups of patients divided according to obesity in terms of gender distribution, age, ECOG performance status, histologic types of the tumor, treatment modality, or PD-L1 expression. Twelve of 29 patients with obesity (41.4%) and 19/89 (21%) patients without received broad-spectrum antimicrobials prior to starting anti-NSCLC therapy (*p* = 0.05). We did not find any significant difference in the OS or PFS between the two groups, nor did we find any difference in the probability of progression of disease despite being started on therapy in both groups. Finally, within the group of patients with MetS, we did not find any difference in the OS among patients who were obese and patients who were not obese (*p* = 0.22).

## Discussion

ICIs, with or without combination chemotherapy, are now the mainstay of therapy for patients with advanced NSCLC that do not carry any actionable driver mutations. However, many patients remain unresponsive to these treatments. ICIs are expensive and carry side-effects. Various studies have been carried out to identify factors that may affect the treatment outcome to help select for the patients who are most likely to benefit from ICI therapy.

In addition to tumor mutation burden (23) and PD-L1 expression levels (24), microsatellite instability (25) and the degrees of CD8 T-cell infiltrate in the tumor microenvironment (26) have also been found to correlate positively with response to ICIs. We and others have previously found that the use of broad-spectrum antimicrobials prior to the initiation of ICI therapy negatively impact the tumor response (27–29). Furthermore, other investigators found that the peripheral blood neutrophil:lymphocyte ratio (NLR) correlated negatively to OS (30). We previously found in NSCLC that higher pre-therapy absolute monocyte counts (AMCs) correlated to shorter time to response but not to the response rate or duration of response (31). We also identified that, although baseline absolute neutrophil counts (ANCs) did not have any prognostic value, ANCs after the first dose predicts for response to ICIs (31).

Obesity is often, but not always, associated with MetS. As shown in our cohort of patients, 41% of the patients with a BMI >30 did not qualify to the diagnosis of MetS. Both obesity and MetS induce ongoing chronic inflammatory processes (8, 20). Chronic inflammation may lower the threshold for the trigger of host immune activation. Since the diagnosis of obesity is based solely on one single clinical parameter of BMI ≥ 30, obesity is a heterogenous disorder with varying degrees of clinical spectrum and, hence, varying intensities of chronic inflammatory processes. It is, therefore, not surprising that studies evaluating the effects of obesity on treatment outcome with ICIs of patients with advanced cancer have yielded mixed results. In contrast, the diagnosis of MetS requires at least three of the five clinical criteria of glucose intolerance, central obesity, hypertriglyceridemia, reduced levels of HDL cholesterol, and hypertension (19). MetS is, therefore, a less heterogenous condition. Because of the differences in the stringency of diagnosis of the two conditions, the effects obesity has on treatment outcome to ICIs in cancer patients may not necessarily mirror those due to MetS. To determine whether these two clinical characteristics confer different effects at an operational level, we carried out a retrospective study of a cohort of patients with advanced NSCLC treated with first-line ICIs.

We identified that MetS is an independent factor that predicts for improved OS in patients with NSCLC treated first-line with ICI monotherapy. The survival advantage in the MetS group was observed despite the fact that a significantly higher proportion of patients with MetS received pre-therapy broad-spectrum antimicrobials that was previously found to adversely affect the ICI treatment outcome (27–29). The survival advantage associated with MetS was, however, only observed in those who received ICI monotherapy and not first-line chemoimmunotherapy and not related to obesity since we did not find any improved outcome when we compared the survival in obese patients with that in non-obese patients. Interestingly, although MetS patients treated with first-line ICI monotherapy enjoyed significantly longer OS, there was no difference in the PFS between the two groups. The reason for the dissociation between OS and PFS is unclear but may suggest that MetS patients were more likely to respond or tolerate second-line therapy (which would invariably be combination chemotherapy) compared to those without MetS, if their disease progressed while being treated with ICIs. An alternate reason is that the ICI may have selected for chemo-sensitive clones in these patients.

Our study suffers the limitations associated with being a retrospective study. Furthermore, being a single-center study, the smaller patient population may not have enough power to detect small differences. Because of this, we could not rule out in our cohort of patients that obesity was not associated with improved outcome. However, being a single-center study, the strengths of this work include patients being treated more uniformly by the same group of medical oncologists, and with similar levels of supportive care provided to the patients. Studies have shown that the level of supportive care may also influence the OS of the patients.

Another limitation in our study is the diagnosis of MetS. Due to the waist circumference of the patients not being available, we adopted a modified criteria that relies on the assumption that a body mass index (BMI) of 30 or higher would satisfy the NIH criteria for large waist given the high correlation between BMI and waist circumferences for either gender (22).

Finally, because the RECIST criteria was not used to evaluate the tumor response outside the context of a clinical trial in our institution, we were unable to determine the effects MetS may have on the response rates using a standardized radiologic measuring method. This led us to the use of Clinical Benefit as the clinical readout.

## Conclusions

In conclusion, this study provides important insights into the possible role of MetS in influencing the OS among patients with advanced NSCLC who received ICI monotherapy as their first-line therapy. A prospective study that involves the use of waist circumference would, therefore, be needed to confirm our findings. If confirmed, baseline MetS should be considered a stratification factor in future ICI clinical trials for NSCLC. Future translational and clinical studies should also include investigations into the immunologic differences between obesity and MetS to provide mechanistic interventional opportunities and improve the treatment outcomes in patients treated with ICI monotherapy for NSCLC.

## Data availability statement

The raw data supporting the conclusions of this article will be made available by the authors, without undue reservation.

## Ethics statement

The studies involving human participants were reviewed and approved by SUNY Upstate Medical University Institutional Review Board. Written informed consent for participation was not required for this study in accordance with the national legislation and the institutional requirements.

## Author contributions

MB, PA, DB, AG, DN, SNi, GG, JL, SNa, BK, MO, FK, BP, JB, MS: Data acquisition. MZ and PA: Coordination. SG and SL: Conceptualization and supervision. SL: Analysis and interpretation. SG and SL: writing, review and editing. All authors contributed to the article and approved the submitted version.
